# Perceptions of personal health risks by medical and non-medical workers in a university medical center: a survey study

**DOI:** 10.1186/1471-2458-10-681

**Published:** 2010-11-09

**Authors:** Tita Alissa Listyowardojo, Raoul E Nap, Addie Johnson

**Affiliations:** 1 Department of Psychology, University of Groningen, Groningen, the Netherlands; 2 University Medical Center Groningen, Groningen, the Netherlands

## Abstract

**Background:**

Health care workers (HCWs) are faced with many work-related choices which may depend on how they perceive risk, such as whether or not to comply with safety regulations. Little research has investigated risk perception in medical workers in comparison with non-medical workers and the extent to which risk perception differs in these groups. The current study thus investigates risk perception of medical and non-medical workers to inform and complement future research on safety compliance. The study has implications for the design of intervention programmes to increase the level of compliance of HCWs.

**Methods:**

A survey study was conducted in which questionnaires were distributed to 6380 HCWs. The questionnaire asked for ratings of risk perception for cold, annual influenza, pandemic influenza, cancer, heart attack and food poisoning. Of 2495 returned questionnaires (response rate: 39%), 61.40% were from medical workers (24.1% of these were from physicians, 39.7% from nurses and 36.2% from paramedics) and 38.60% were from non-medical workers.

**Results:**

Medical workers gave lower risk perception ratings than did non-medical workers for cancer, but not for other health risks. Within the medical workers, physicians rated the risk of getting a cold as higher, but of having a heart attack as lower than did nurses and paramedics; physicians also rated their risk of getting cancer as lower than did nurses. Perceived risk was higher as a function of age for pandemic influenza, cancer and heart attack, but lower for cold and annual influenza. HCWs who lived with a partner and children rated the risk of getting a cold or annual influenza higher than those who lived alone or with a partner only. Full-time HCWs gave lower ratings for annual influenza than did part-time HCWs.

**Conclusions:**

Different base levels of risk perception between medical and non-medical workers need to be taken into account for successful implementation of safety regulations.

Intervention programmes to improve compliance with safety regulations may need to be customized for different groups as a function of how they perceive risk.

## Background

HCWs are faced with work-related choices such as whether to participate in voluntary immunization programmes or to comply with safety regulations. Compared to non-medical workers who have limited contact with infected patients, medical workers are exposed to various occupational health hazards which can result in serious long-term adverse health outcomes [1-3]. This makes compliance with safety regulations especially important for medical workers' personal health. Previous studies have reported low rates of compliance of HCWs with hospital regulations and recommendations such as universal precautions (UPs) [4,5] and vaccination programmes [6]. The extent to which HCWs comply with safety regulations is likely to be related to their perceptions of the personal risks involved with the behaviours being regulated [7,8]. For example, compliance with UPs is lower among those who rate their personal risk of infection lower [4], and HCWs who perceive their risk of contracting an infection as higher are also more likely to participate in a pre-pandemic influenza vaccination programme than those who perceive their risk as lower [9].

Previous work on compliance has focused primarily on HCWs' perceptions of occupational risks such as influenza [6,10-12] and those related to exposure to blood-borne viruses [13], and not on their perceptions of general health risks such as heart attack. Understanding how HCWs perceive health-related risks, in general, can help to understand factors involved in compliance with safety regulations. Research on risk perception has shown that people tend to rate their own personal risk related to general health conditions lower than they rate risks for others [8]. This is a concern because if people are unrealistically optimistic about their health, they will tend to feel less susceptible to diseases and be less likely to change their behaviour to reduce risks by [14], for example, complying with safety regulations.

The current research compared the ratings of perceived risk of medical and non-medical workers to draw conclusions that may inform future research on compliance and risk perception. The study has significant implications for the design of intervention programmes to increase the level of compliance with safety regulations for different groups (i.e., medical and non-medical workers).

## Methods

### Participants

Stratified sampling across professional groups was used to select medical (i.e., physicians, nurses and paramedics) and non-medical (e.g. financial services, board of directors, human resource management) workers of the University Medical Center Groningen (UMCG), the Netherlands, for invitation to participate in the study. The UMCG has approximately 1,300 beds, including 53 surgical and medical adult intensive care beds and 46 neonatal and pediatric intensive care beds. The UMCG is the only university medical center in the northern part of the country and as such is the major hospital of referral for patients with many types of illness. In October and November 2008, invitations to participate in an on-line survey were sent electronically to medical and non-medical workers in the group of interest. Because the participants of the study were not patients and the study was conducted anonymously and based on voluntary participation, approval of the medical ethical committee was not necessary.

### Questionnaire

The questionnaire used was administered as a part of a larger study about compliance of HCWs with guidelines for controlling pandemic influenza [15]. The demographic information asked for in the questionnaire included function in the hospital (medical or non-medical worker, physician or nurse or paramedic for medical workers), gender, age, type of work contract (i.e., full-time or part-time) and family status (i.e., lives alone or with a partner and/or children). The risk perception questions were of the form "What is the likelihood that you will have or get ... in the next one year?" This question was completed with "a cold", "annual influenza", "pandemic influenza", "cancer", "a heart attack" and "food poisoning". Responses were made using a Likert scale ranging from 1 (very unlikely) to 5 (very likely).

### Statistical Analyses

Differences in demographic characteristics between medical and non-medical workers and between roles within the medical group (physicians, nurses and paramedics) were tested using Pearson's chi-square tests, except for age, which was tested using a *t*-test (for medical vs. non-medical groups) and one-way ANOVA (within medical group). ANCOVAs were conducted to determine whether demographic variables interacted with group and role to determine risk perception. Separate ANCOVAs were conducted for each of the health risks. A significance level of *p *< .05, Bonferroni corrected for multiple comparisons where necessary, was used for all analyses.

## Results

A total of 2495 questionnaires were returned out of the 6380 questionnaires sent, for a response rate of 39%. Of the returned questionnaires, 61.40% were from medical workers (*n *= 1532) and 38.60% were from non-medical workers (*n *= 963). Within the medical workers (*n *= 1532), 369 (24.1%) respondents were physicians, 608 (39.7%) were nurses and 555 (36.2%) were paramedical health care workers. The demographic characteristics are presented in Table 1.

**Table 1:** Demographic characteristics of health care workers by group and role

	Medical group				
			
Category	Physicians (*n *= 369)	Nurses (*n *= 608)	Paramedics (*n *= 555)	Total Medical group (*n *= 1532)	Total Non- medical group (*n *= 963)
Age (mean, with standard deviation in parentheses)	39.06 (11.01)	42.18^a^** (10.47)	42.67^a^** (10.56)	41.61 (10.72)	44.25^b^** (10.03)

Gender (%)					
Male	53.39	19.57	26.85	30.35	36.66
Female	46.61	80.43^c^**	73.15^c^**	69.65^c^**	63.34^c^**

Family status (%)					
Live alone	23.58	18.09	18.20	19.45	18.38
Live with a partner only	35.23	31.58	31.53	32.44	33.96
Live with children only	2.71	2.80	4.14	3.26	5.19
Live with a partner and children	38.48	47.53	46.13	44.84	42.47

Type of work contract (%)					
Full-time (40 hrs/week)	75.61^d^**	31.74	41.98	46.02	50.88
Part-time (mean = 26.5 hrs/week; *SD *= 7.5 hrs/week)	24.39	68.26^e^**	58.02^e^**	53.98^e^*	49.12

^a^Significantly older than physicians. ^b^Significantly older than medical group. ^c^Significantly outnumbered male counterparts. ^d^Significantly outnumbered part-timers. ^e^Significantly outnumbered full-timers. **p < .001. *p < .01.

The average age was 42.63 (*SD *= 10.54) years old. Non-medical workers were older than were medical workers (*t*(2142.77) = 6.23, *p *< .001; see Table 1). Within the medical group, one-way ANOVA conducted on age with role (i.e., physicians, nurses, paramedics) as a between-subject factor revealed a main effect of role (*F*(2, 1526) = 14.19, *p *< .001; see Table 1). Chi-square analyses revealed interactions between gender and group (medical vs. non-medical workers; *X*^2 ^(1, *n *= 2495) = 10.66, *p *< .05; see Table 1) and between gender and role within the medical group (*X*^2 ^(2, *n *= 1532) = 129.27, *p *< .001; see Table 1). Chi-square analyses also revealed interactions between type of work contract and group (*X*^2 ^(1, *n *= 2495) = 5.61, *p *< .05; see Table 1) and between type of work contract and role (*X*^2 ^(2, *n *= 1532) = 183.59, *p *< .001; see Table 1). No significant differences were found for family status between medical and non-medical groups or between physicians, nurses and paramedics.

The mean risk perception ratings of the medical and non-medical workers are given in Table 2. To investigate whether demographic characteristics interacted with group to determine risk perception, ANCOVAs were conducted with age as a covariate and group (medical vs. non-medical), gender (female vs. male), type of work contract (full-time vs. part-time) and family status (live alone, live with a partner only, live with children only, live with a partner and children) as between-subject variables. The ANCOVAs showed that age as the covariate was significantly related to risk perception for all health risks except for food poisoning. Parameter estimates showed that risk perception increased with age for pandemic influenza (*β *= .01, *p *< .001), cancer (*β *= .02, *p *< .001) and heart attack (*β *= .03, *p *< .001), but decreased with age for cold (*β *= - .02, *p *< .001) and annual influenza (*β *= - .01, *p *< .001). The analyses showed a main effect of group for risk perception for cancer (*F *(1, 2458) = 5.65, *p *< .05; see Table 2). No other main effects of group were significant. The main effect of type of work contract was significant only for annual influenza (*F *(1, 2458) = 4.89, *p *< .05). Full-time workers rated the risk for annual influenza lower than did part-time workers (mean = 2.40, SE = .05 vs. mean = 2.57, SE = .06, *p *< .05). The main effect of family status was significant for cold (F (3, 2458) = 9.47, *p *< .001) and annual influenza (F (3, 2458) = 4.34, *p *< .01). HCWs who lived alone rated the risk for cold lower than did HCWs who lived with a partner and children (mean = 3.69, SE = .07 vs. mean = 4.03, SE = .05, *p *< .001). HCWs who lived with a partner only rated the risk for annual influenza lower than did HCWs who lived with a partner and children (mean = 2.39, SE = .04 vs. mean = 2.58, SE = .04, *p *< .01).

**Table 2:** The mean risk perception ratings by group and role for each health risk (standard error of the mean in the parentheses)

Risk perception	Physicians (*n *= 369)	Nurses (*n *= 608)	Paramedics (*n *= 555)	Total medical workers (*n *= 1532)	Total non- medical workers (*n *= 963)
For cold	4.23 ( .05)	3.77a*** ( .05)	3.86^a^* ( .05)	3.91 ( .03)	3.79 ( .04)

For annual influenza	2.45 ( .05)	2.43 ( .04)	2.39 ( .04)	2.42 ( .02)	2.51 ( .03)

For pandemic influenza	1.49 ( .04)	1.73 ( .03)	1.63 ( .03)	1.64 ( .02)	1.66 ( .03)

For cancer	1.41 ( .04)	1.83^b^** ( .03)	1.79 ( .04)	1.72 ( .02)	1.89^c^* ( .03)

For heart attack	1.34 ( .03)	1.69^b^** ( .03)	1.68^b^** ( .03)	1.60 ( .02)	1.78 ( .03)

For food poisoning	2.12 ( .05)	2.16 ( .04)	2.14 ( .04)	2.14 ( .02)	2.14 ( .03)

^a^Significantly lower than physicians' ratings. ^b^Significantly higher than physicians' ratings. ^c^Significantly higher than medical workers' ratings. ***p < .001. **p < .01. *p < .05.

ANCOVAs were also conducted within the medical group with age as a covariate and role (physician, nurse or paramedic), gender (female vs. male), type of work contract (full-time vs. part-time) and family status (live alone, live with a partner only, live with children only, live with a partner and children) as between-subject variables. The ANCOVAs showed that age as a covariate was significantly related to all health risks, except for food poisoning. Parameter estimates showed that risk perception increased with age for pandemic influenza (*β *= .01, *p *< .01), cancer (*β *= .01, *p *< .001) and heart attack (*β *= .02, *p *< .001), but decreased with age for cold (*β *= - .02, *p *< .001) and annual influenza (*β *= - .01, *p *< .001). The analyses showed a main effect of role on risk perception for cold (*F *(2, 1482) = 5.81, *p *< .01), cancer (*F *(2, 1482) = 4.37, *p *< .05) and heart attack (*F *(2, 1482) = 6.39, *p *< .01; see Table 2). The main effect of family status was significant for cold (F (3, 1482) = 6.19, *p *< .001) and annual influenza (F (3, 1482) = 2.77, *p *< .05). Posthoc tests using Bonferroni correction showed that medical workers who lived with a partner and children gave higher ratings to the risk of getting a cold (mean = 4.08, SE = .09 vs. mean = 3.70, SE = .07, *p *< .001**) **or an annual influenza (mean = 2.51, SE = .05 vs. mean = 2.29, SE = .06, *p ***<**.05) than did medical workers who lived with a partner only.

## Discussion

Compliance with safety regulations can be explained, at least in part, by how HCWs perceive risks. If perceived risk is low, the incentive to comply may be lacking. In this study we looked for and found differences in how different groups of HCWs perceive risk. Most importantly, both type of function and demographic characteristics of HCWs were found to influence risk perception.

### Effects of function type

Medical workers perceived their risk of getting cancer as lower than did non-medical workers. The lower risk perception for cancer of medical workers is largely due to the relatively low ratings given by physicians. A number of factors may contribute to the lower risk ratings of physicians for cancer. The fact that physicians may have to diagnose cancer patients and are directly involved in treating them may lead physicians to feel that they have more control over cancer than do nurses and paramedics. Both this perceived control and familiarity with risk may influence their risk perception [16]. It is also possible that physicians define risks differently than do nurses and paramedics. The current study found that physicians tend to perceive their risks related to more serious health risks (i.e., cancer and heart attack) as lower than do nurses and paramedics, but those related to a less serious health risk (i.e., cold) as higher. Physicians may define risk based on the probability of occurrence of the health hazard, whereas nurses and paramedics may be influenced by the severity of the disease in their perceptions of risk [17]. That is, physicians may have been more able than nurses or paramedics to do what was asked in this study, namely to rate the likelihood of suffering a health condition in the next year. Finally, the fact that physicians may need to communicate health risks more often to patients (or the general public) than do nurses and paramedics [18,19] may influence how physicians perceive health risk. For example, the expectation for physicians to sympathize with patients' conditions in communicating risk [20] may lead physicians to slightly emphasize the benefits of medical treatments and minimize the severity of serious health risks [18].

### Effects of age and family status

The current study also found that aging is correlated with higher risk perception for pandemic influenza, cancer and heart attack but lower risk perception for annual influenza and cold. HCWs are probably fully aware that aging is correlated with increased health risks such as cancer [21], cardiovascular disease [22] and pandemic influenza [23], making it unsurprising that these risks are rated higher by older HCWs. Younger HCWs may realize that they are not in the risk group of getting or having cancer, cardiovascular disease and pandemic influenza, thus leading to lower ratings in this age group.

Age tends to be confounded with family status, with younger workers being more likely to live with children. Given that children who still live with their parents may be young and susceptible to cold and annual influenza, it stands to reason that HCWs who live with a partner and young children rate their chances of contracting a cold or annual influenza higher than those who live alone or with a partner only.

Our findings of decreased risk perception with increasing age for annual influenza should be interpreted with caution considering the lack of vaccination status data in this study (the overall uptake rate for influenza vaccination at the UMCG in the years 2006-2009 was 21% to 34%). If the older HCWs were vaccinated for annual influenza or were planning to be vaccinated, this could lead them to rate their risk as lower.

### Limitation

The relatively low response rate of 39% is the main limitation of the study. Although the number of participants in each group in the study fits the profile of the target population, we cannot preclude non-response bias.

## Conclusions

Different base levels of risk perception between medical and non-medical workers and among medical workers need to be taken into account for successful implementation of safety regulations. Intervention programmes to improve compliance with safety regulations may need to be customized for different groups as a function of how they perceive risk.

## Competing interests

The authors declare that they have no competing interests.

## Authors' contributions

TAL contributed to study conception and design, acquisition of data, data analysis and interpretation, drafting and critically revising the manuscript. REN contributed to study conception and design, acquisition of data and critically revising the manuscript. AJ contributed to data analysis and interpretation, and drafting and critically revising the manuscript. All authors read and approved the final manuscript.

## Pre-publication history

The pre-publication history for this paper can be accessed here:

http://www.biomedcentral.com/1471-2458/10/681/prepub
